# Clinical Effectiveness of Peramivir in Comparison with Other Neuraminidase Inhibitors in Pediatric Influenza Patients

**DOI:** 10.1155/2012/834181

**Published:** 2012-02-22

**Authors:** Toshiyuki Hikita, Hiroyuki Hikita, Fusako Hikita, Naoko Hikita, Shizue Hikita

**Affiliations:** Pediatric Division, Hikita Pediatric Clinic, 2-7-20 Nakamachi, Kiryu City, Gunma 376-0035, Japan

## Abstract

The currently used antivirals in the treatment of influenza in Japan include amantadine, oseltamivir, zanamivir, laninamivir, and peramivir. We compared the efficacy of intravenous peramivir with that of other neuraminidase inhibitors for treating pediatric influenza. The present study included 223 influenza patients (≤18 years) who presented at the Hikita Pediatric Clinic between February and April 2011. We compared fever duration after starting treatment with antiviral drugs. Because inhalation drugs are difficult to use in <5-year-old patients and because of the potential adverse effects of oseltamivir in teenagers, we created two different age groups (<10-year-old group and 5–18-year-old group) to evaluate treatment results. In influenza A patients between 5 and 18 years old, the median fever duration after treatment with zanamivir was 2 days, compared with 1 day for peramivir (*P* = 0.0242). In influenza B patients between 5 and 18 years old, the median fever duration after treatment with laninamivir was 3 days, compared with 1 day for peramivir (*P* = 0.0097). We found no significant difference for any of the other combinations of drug/disease type/age groups. No adverse effects were observed with the antiviral drugs used. The results suggest that peramivir is very useful in pediatric influenza patients.

## 1. Introduction

Of the various respiratory diseases, influenza is a major cause of mortality and morbidity among patients, particularly the very young and the elderly [1, 2]. Two options are available for moderating the effect of the influenza virus: vaccines, which although effective, are underutilized and not completely protective because of frequent antigenic shifts in the viral surface proteins and antiviral drugs [2, 3]. Antiviral drugs have emerged as attractive options in the battle against influenza. Amantadine, oseltamivir, zanamivir, laninamivir, and peramivir are the five antiviral drugs currently used to treat influenza in Japan [2]. However, firm guidelines for prescribing these drugs remain to be established.

Amantadine is limited in effectiveness because of its lack of activity against influenza B virus [4] and the rapid emergence of resistant viral strains. Hemagglutinin and neuraminidase, two glycoproteins present on the viral surface, have antiviral targets [5]. Recently, oseltamivir and zanamivir, two influenza neuraminidase inhibitors, have commonly been prescribed for influenza A and B [6–12]. Laninamivir is a long-acting neuraminidase inhibitor for the treatment of influenza. A single inhalation of laninamivir is effective for the treatment of influenza, including that caused by the oseltamivir-resistant viruses, in adults [13, 14]. However, seriously ill and pediatric patients need a parenteral formulation because the injectable drug is much easier to administer in such cases than oral oseltamivir, inhaled zanamivir, or laninamivir.

In Japan, peramivir has recently been approved for use not only in adults but also in children over 1 month of age [15]. In this study, we compared the efficacy of intravenous peramivir with that of other neuraminidase inhibitors for treating influenza infections in pediatric patients.

## 2. Material and Methods

The present study included 223 patients under the age of 18 years diagnosed with influenza at the Hikita Pediatric Clinic between February and April 2011. The patients presented with a complaint of fever lasting for less than 48 h, and they were clinically diagnosed with rapid diagnostic tests. Specimens from nasal swabs or nasal aspirates were subjected to antigen detection. Commercial antigen detection kits based on immunochromatography (The Quick Chaser Flu A, B rapid antigen test [Mizuho Medy Co., Ltd. Saga, Japan]) was used for the diagnosis of influenza A or B. Subsequently, after obtaining informed consent from the parents, 35 patients diagnosed with influenza A by the rapid antigen A, B test underwent a 2009 influenza A H1N1 virus infection test using the Quick Chaser Flu AH1pdm (Mizuho Medy) to differentiate patients with 2009 H1N1 influenza from those with seasonal influenza.

 The efficacy, potential adverse effects, and convenience of administration of the five antiviral drugs were explained to the patients and/or their families prior to study initiation. The choice of antivirals for influenza treatment was then discussed, and after obtaining informed consent from patients and/or their families, all patients underwent antiviral therapy. Laninamivir was administered as a single-inhalation dose of 40 mg for patients aged ≥10 years or 20 mg for patients aged <10 years. Peramivir was administered intravenously as a single dose of 10 mg/kg/dose (maximum 300 mg/dose) over a period of 15 min. Oral oseltamivir was prescribed twice per day in divided dosages for 5 days (4 mg/kg/day, maximum of 150 mg/day). Zanamivir was administered twice per day at an inhalation dosage of 20 mg/day for 5 days.

In 2007, the Ministry of Health, Labor, and Welfare, Japan, issued emergency instructions suspending the use of oseltamivir in patients aged 10–19 years [16]. Accordingly, oseltamivir was not prescribed for the teenage patients with influenza in our study. In addition, inhaled drugs are difficult to use in infants. Therefore, laninamivir and zanamivir were prescribed for patients aged ≥5 years. We created 2 age groups for statistical analysis: patients aged <10 years and those aged between 5 and 18 years. We compared the duration of fever after initiating antiviral therapy. Fever was considered positive if the patient's body temperature was ≥37.5°C. When a patient's body temperature dropped and remained at <37.5°C for 48 continuous h, the fever was considered as resolved.

 Data were analyzed using JMP software version 8.0.2 (SAS Institute, Cary, NC, USA). The Wilcoxon rank-sum test and/or the Kruskal-Wallis rank-sum test were used to compare the ages of influenza patients treated with laninamivir, oseltamivir, peramivir, or zanamivir. We used Kaplan-Meier analysis and the log-rank test to compare the duration of fever after antiviral therapy initiation. A *P* value of <0.05 was considered statistically significant.

## 3. Results

We treated 131 (70 males) and 92 (53 males) patients with influenza A and B, respectively. Nine of 35 (25.7%) patients tested positive in the 2009 influenza A H1N1 test. Patients were administered the following antiviral drugs: laninamivir (*n* = 14), oseltamivir (*n* = 125), peramivir (*n* = 45), and zanamivir (*n* = 39). No patient was treated with amantadine in this study. The median ages of the patients treated with laninamivir, oseltamivir, peramivir, and zanamivir were 132.5 months (range, 80–164), 56.5 months (range, 3–135), 76 months (range, 2–219), and 124.5 months (range, 59–198), respectively. No adverse effects were observed with any of the antiviral drugs used in this study.

We compared <10-year-old influenza patients who were administered either oseltamivir or peramivir (Table 1). The age of patients treated with oseltamivir was not significantly different from that of patients treated with peramivir. We also compared 5–18-year-old influenza patients treated with peramivir, zanamivir, or laninamivir. The age of patients did not significantly differ between treatment groups. The median duration of fever after zanamivir treatment in 5–18-year-old patients with influenza A was 2 days (range, 0–3 days), whereas that after peramivir treatment was 1 day (range, 1-2 days); this difference was statistically significant (*P* = 0.0283). The median duration of fever after laninamivir treatment in 5–18-year-old patients with influenza B was 3 days (range, 1–5 days), whereas that after peramivir treatment was 1 day (range, 1–4 days); this difference was also statistically significant (*P* = 0.0097). No other significant differences were observed for any of the other drug/disease type/age group combinations.

## 4. Discussion

Hernandez et al. reported their clinical experience with children (*n* = 11) hospitalized for 2009 influenza A (H1N1) and treated with peramivir [17]. In their study, all patients had rapidly progressing, radiographically confirmed viral pneumonia with respiratory failure. In our study, peramivir was administered to patients with influenza, including 2009 influenza A (H1N1), seasonal influenza H3N2, and influenza B. None of the patients had severe disease and all were treated as outpatients. Severe adult influenza infection has been reportedly treated with peramivir [18]. Although oseltamivir and zanamivir are used for severe influenza infections such as encephalopathy [19], peramivir is more suitable for such infections because it is much easier to administer (intravenously) to a severe case when compared with oral oseltamivir or inhaled zanamivir. Currently, most patients in Japan with an influenza-like illness are tested using rapid diagnostic tests and treated with an appropriate choice of antiviral drugs if results prove positive [20]. Particularly, after the 2009 H1N1 influenza pandemic virus was isolated, antiviral therapy was recommended for all pediatric influenza patients.

 A limitation of this study was the small study sample and the lack of a randomized open label study; therefore, more data are needed before the clinical implications of this study become clear. We compared only fever duration and did not analyze any other symptoms because influenza patients in Japan can only return to school 48 hours after fever resolution. However, we were able to confirm the usefulness of peramivir in children. Randomized case-control studies with sufficiently large populations will be conducted during the next influenza season following approval by an ethics committee.

 Recent developments in antigen detection tests have made it possible to differentiate between influenza A, 2009 influenza A (H1N1), and influenza B. Therefore, on determination of the variant involved, physicians, patients, and their families can make informed decisions regarding choice of antiviral drug. The inhalation drugs laninamivir and zanamivir are difficult to use in infants. Vaccines are not completely protective because of frequent antigenic shifts in the viral surface proteins, as observed in the last pandemic.

 The results of this study suggest that peramivir is an important and a feasible therapeutic option for pediatric influenza patients.

## Figures and Tables

**Table 1:** Comparison of the effectiveness of oseltamivir, zanamivir, laninamivir, and peramivir against influenza virus infection.

Groups		Therapy	Number of patients	Median age in months (range)	Duration of fever before treatment, median day (range)	Duration of fever after treatment, median day (range)	*P* value
Influenza type	Ages (years)						
Influenza A	0–9	Oseltamivir	83	51 (3–118)	1 (0–2)	2 (0–6)	0.4499
		Peramivir	22	41.5 (2–106)	1 (0–2)	1.5 (1–3)	

Influenza B	0–9	Oseltamivir	41	81 (25–118)	1 (0–2)	2 (0–4)	0.6435
		Peramivir	13	75 (10–118)	0 (0–2)	2 (1–4)	

Influenza A	5–18	Laninamivir	1	80 (80-80)	0 (0-0)	1 (1-1)	Not performed
		Peramivir	15	94 (72–219)	1 (0–2)	1 (1-2)	
		Zanamivir	18	34.5 (69–198)	1 (0–2)	2 (0–3)	0.0242*

Influenza B	5–18	Laninamivir	13	134 (91–164)	0 (0–2)	3 (1–5)	0.0097*
		Peramivir	13	98 (63–167)	1 (0-1)	1 (1–4)	
		Zanamivir	20	120 (87–179)	0 (0–2)	2 (0–4)	0.2979

“Not performed” indicates that statistical analysis was not performed because the number of subjects was too small.

*indicates a significant difference between peramivir and the other administered drug.

(Both the 0–9 years group and the 5–18 years group included 5–9-year-old children treated with peramivir).
